# Remote Monitoring of Colorectal Cancer Survivors Using a Smartphone App and Internet of Things–Based Device: Development and Usability Study

**DOI:** 10.2196/42250

**Published:** 2023-02-15

**Authors:** Seyed Mohammad Ayyoubzadeh, Tayebeh Baniasadi, Mohammad Shirkhoda, Sharareh Rostam Niakan Kalhori, Niloofar Mohammadzadeh, Kamran Roudini, Reza Ghalehtaki, Fereidoon Memari, Amirmohsen Jalaeefar

**Affiliations:** 1 Department of Health Information Management School of Allied Medical Sciences Tehran University of Medical Sciences Tehran Iran; 2 Department of Health Information Technology Faculty of Para-Medicine Hormozgan University of Medical Sciences Bandar Abbas Iran; 3 Department of General Surgery, Subdivision of Surgical Oncology Cancer Institute of Iran Tehran University of Medical Sciences Tehran Iran; 4 Peter L. Reichertz Institute for Medical Informatics (PLRI) Technical University of Braunschweig and Hannover Medical School Braunschweig Germany; 5 Department of Internal Medicine School of Medicine Tehran University of Medical Sciences Tehran Iran; 6 Department of Radiation Oncology, Cancer Institute Radiation Oncology Research Center, Cancer Research Institute Tehran University of Medical Sciences Tehran Iran

**Keywords:** eHealth, telemedicine, colorectal cancer, cancer survivor, IoT, mHealth, patient monitoring, remote monitoring, postdischarge care, cancer, patient care, cancer care, postoperative complications

## Abstract

**Background:**

Patients with colorectal cancer who undergo surgery face many postoperative problems. These problems include the risk of relapse, side effects, and long-term complications.

**Objective:**

This study sought to design and develop a remote monitoring system as a technological solution for the postdischarge care of these patients.

**Methods:**

This research was conducted in 3 main steps: system feature extraction, system design, and evaluation. After feature extraction from a systematic review, the necessary features were defined by 18 clinical experts in Iran. In the next step, the architecture of the system was designed based on the requirements; the software and hardware parts of the system were embedded in the architecture, then the software system components were drawn using the unified modeling language diagrams, and the details of software system implementation were identified. Regarding the hardware design, different accessible hardware modules were evaluated, and suitable ones were selected. Finally, the usability of the system was evaluated by demonstrating it over a Skype virtual meeting session and using Nilsen’s usability principles.

**Results:**

A total of 21 mandatory features in 5 main categories, including patient information registration, periodic monitoring of health parameters, education, reminders, and assessments, were defined and validated for the system. The software was developed using an ASP.Net core backend, a Microsoft SQL Server database, and an Ionic frontend alongside the Angular framework, to build an Android app. The user roles of the system included 3 roles: physicians, patients, and the system administrator. The hardware was designed to contain an Esp8266 as the Internet of Things module, an MLX90614 infrared temperature sensor, and the Maxim Integrated MAX30101 sensor for sensing the heartbeat. The hardware was designed in the shape of a wristband device using SolidWorks 2020 and printed using a 3D printer. The firmware of the hardware was developed in Arduino with the capability of firmware over the air. In evaluating the software system from the perspective of usability, the system received an average score of 3.8 out of 5 from 4 evaluators.

**Conclusions:**

Sensor-based telemonitoring systems for patients with colorectal cancer after surgery are possible solutions that can make the process automatic for patients and caregivers. The apps for remote colorectal patient monitoring could be designed to be useful; however, more research regarding the developed system’s implementation in clinic settings and hospitals is required to understand the probable barriers and limitations.

## Introduction

Cancer is one of the leading causes of death worldwide. According to reported statistics by the World Health Organization in 2021, this disease was the cause of nearly 10 million deaths worldwide [1].

Among the types of cancer, colorectal cancer (CRC) has the highest incidence of new gastrointestinal cancers globally. CRC includes the colon and rectal cancers [2]. New CRC cases are 19.7 per 100,000 people globally and 12.9 in Iran [3]. In a review conducted in 2019, CRC was reported to be one of the most common cancers among Iranian men and women in the whole review investigation period (2004-2009) [4]. In 2020, CRC accounted for 10% (1.9 million cases) of global cancer incidence and 9.4% (0.9 million deaths) of cancer deaths. CRC is the third-most deadly cancer in both genders worldwide. The international number of new CRC cases, based on population growth, aging projection, and human development, is predicted to reach 3.2 million in 2040 [5].

Strategies of treatment for CRC vary according to the stage and type of cancer. Some treatment procedures include endoscopy for macroscopic intramucosal carcinoma, surgical lymph node dissection, laparoscopic surgery, palliative chemotherapy, radiotherapy, extensive surgery, and local ablative therapies for metastases [5,6].

In the meantime, CRC surgery is associated with many different complications that affect the efficacy of the surgery and patients’ overall health and survival [6]. The most frequent postoperative surgical complications after colorectal resections are surgical site infection, anastomotic leakage, intra-abdominal abscess, ileus, and bleeding. These complications have different influences on the outcomes and have to be diagnosed accurately. Monitoring and standardization of postoperative care to minimize these complications are essential [7].

Most of these complications usually occur in the first week [8] to the first month [9] after surgery. Therefore, these patients need continuous care during this period. Due to the lack of specialized personnel and their high workload [10], on the one hand, and the high costs of health care for patients with cancer [11], on the other, the importance of technology-based intervention to monitor the condition of surgical patients after discharge is increasing.

In addition, the 5 most common factors in admitting patients with cancer to the intensive care unit include sepsis, respiratory failure, heart failure, cardiopulmonary resuscitation, and surgical complications [12]. Monitoring the patients could help early detection of these complications. Moreover, patient monitoring can give the physician a clear vision of the discharged patient’s health status. If patients are not monitored and followed after discharge, different events may occur, including emergency conditions, unplanned readmission to the intensive care unit, unplanned resurgery, or specific complications such as infection [13].

Telemedicine services have become a powerful solution for providing health services. Studies show the impact of telemedicine services on time savings, patient transportation, and cost savings. The use of these technologies can satisfy patients and health care providers and facilitate their affairs [14]. Since it has been estimated that patients tend to use mobile apps in the postsurgery period [15], it is possible to create a platform to facilitate communication between the patient and the care team using mobile technologies [16], especially smartphones. Health care providers make decisions based on laboratory tests, reports, and self-reported data and according to the patient’s symptoms [17]. So, mobile apps have been developed to monitor patients’ postdischarge and recovery duration [17,18].

In this regard, similar previous studies were accomplished for remote care, self-management, and telemonitoring of patients with cancer after surgery by applying telephone calls, messaging systems, web portals, and mobile apps [19-21].

Also, more specific studies have been carried out for postoperative telemonitoring, education, and self-care in CRC [22-25]. For example, Keng et al [26] developed an integrated discharge monitoring system based on a mobile app to support patients at home after colorectal surgery. Their study included 106 participants, and 93 of them used the designed apps. Another study by Miller et al [22] developed a remote monitoring application to support and improve the care of patients with CRC for the first 30 postoperative days. Their study included 9 clinicians and 10 patients in phase 1 of their study, which was conducted to identify the views of patients and clinicians regarding the remote monitoring app. Phase 2, which included 15 clinicians and 8 patients, was conducted to evaluate the views and usability of a paper-based version of the app. Sun et al [23], in a pilot study, developed a wireless outcomes monitoring program for major abdominal cancer surgery. The study evaluated their system on 20 patients. In a recent study by Salmani et al [25], a smartphone-based app for the self-management of patients with CRC was developed. In another former study, Kim et al [24] developed and assessed a mobile web-based educational program for patients with CRC undergoing enhanced recovery after surgery. In their study, 59 colorectal patients were assigned to the treatment group that received mobile health intervention, and 59 patients were assigned to the conventional care group.

Despite the research conducted on remote monitoring apps for CRC survivors, there has still been a research gap in the development of a system for telemonitoring patients with CRC after surgery equipped with sensors that can collect the data on time and give the patients suitable messages based on the situation. These multiuser mobile-based monitoring systems could provide the ability to collect, analyze, and give proper feedback to both patients and health care providers simultaneously.

Due to the issues raised and the lack of electronic systems for remote monitoring of patients with CRC in Iran, there is a need to develop such a monitoring system. Therefore, the study’s purpose is to design and evaluate a remote monitoring system for patients with CRC undergoing surgery.

## Methods

### Ethics Approval

The clinician experts evaluated the proposed software system for proof of concept; no patient data were used in this study. The Research Ethics Committees of the School of Public Health and Allied Medical Sciences, Tehran University of Medical Sciences approved the current research ethics with the approval ID IR.TUMS.SPH.REC.1399.270.

### System Development

In the first step, the requirements of such a system were gathered from the literature and the opinions of experts. This step is explained in detail in our previous research [27]. After this step, a system architecture containing software and hardware was designed to fulfill the requirements. The software and hardware were developed based on the available technologies and tools.

### Software Design

To design the software, use case diagrams are designed and evaluated for this system. After this step, the suitable tools to create the software systems were chosen, and the software was created. The REST (representational state transfer) architecture was used to develop the web service, and the PWA (progressive web application) approach was used to enable the software to run offline using cached data.

Due to the diversity of users’ devices (Android [Google Inc]–based and iOS [Apple Inc]–based phones) for developing client-side software, the Ionic software development kit on the Angular framework was used to develop a mobile hybrid app. After the client-side programming (in Visual Studio Code v1.52 [Microsoft]), the outputs were generated as PWA and Android-based software. The output of the Java code was generated and then compiled by the Android Studio 4.1.1 Integrated Development Environment (IDE).

### Hardware Design

Due to the need for hardware customization, hardware was designed and created. The hardware is designed to be a wristband with the capability of sensing the heartbeat and body temperature. For this purpose, photoplethysmography (PPG) sensors, temperature sensors, Internet of Things (IoT) modules, batteries, and display modules were selected among the available options. To select the appropriate PPG sensor, due to the elimination of ambient noise and higher accuracy requirements, MAX30101, a ready-made module, was selected. Next, an IoT module, the Wemos D1 mini development board (based on the ESP8266mod), was selected based on its appropriate capabilities and price. An MLX90614 infrared thermometer is used as the temperature sensor. Other components, such as the battery and the display, were chosen in the next step. After selecting the hardware modules, the hardware prototype circuit was created on a breadboard. The firmware was developed in Arduino and then finalized by designing and printing the circuit on a printed circuit board. A wristband enclosure for the board is designed in SolidWorks 2019 software (SolidWorks Corp) and printed using a 3D printer.

### System Usability Evaluation

After creating the system, to perform usability evaluation, explanations of the system were provided to 4 experts (this number corresponds to the number of evaluators (3 to 5 people) proposed by Nielsen [28]) in a virtual session through Skype software (Skype Technologies, a division of Microsoft). By providing the username and password to log in to the system, they were asked to evaluate the system’s usability by completing an online questionnaire. This questionnaire was designed based on Nielsen’s 10 principles [29]. Finally, 4 experts evaluated the system.

## Results

### System Development

The set of eHealth system capabilities related to patients with CRC and survivors obtained from the categorization of requirements is given in Textbox 1.

**The set of eHealth system capabilities related to colorectal cancer patients and survivors.**

**Patient information registration**

- Registration of patient social and demographic information
- Registration of the details of diagnosis and preoperative treatments
- Registration of surgical specifications and postoperative treatments

**Periodic monitoring of health parameters**

- Weight monitoring
- Side effects monitoring

**Education**

- Cancer information
- Common issues and problems for colorectal cancer patients and survivors
- Information about medication
- Information about chemotherapy
- Nutrition information
- Information about rehabilitation
- Information about the treatment process
- Informing about postdischarge care
- Information on pain management
- Information on emergency management

**Reminders**

- Reminders of hospital referrals
- Reminder for drug use
- Patient-tailored information

**Assessments**

- Quality of life assessment
- Nutrition status assessment
- Physician-patient relationship assessment

According to the expected capabilities of the system, the general architecture of the system consists of 3 software parts: client-side application, web service, and database. A hardware part containing a smart wristband has also been embedded in the architecture (Figure S1 in Multimedia Appendix 1).

### Software Design

The Microsoft Visual Studio 2019 IDE and Microsoft SQL Server 2019 were used to develop server-side software (back end) and database, respectively. The web services were implemented with the ASP.NET Core framework (Microsoft).

The Visual Studio Code v1.51.1 IDE was used to develop the client-side software (front end). As a mobile hybrid app, Ionic software development kit and Angular framework were used for software development.

**Figure 1:**
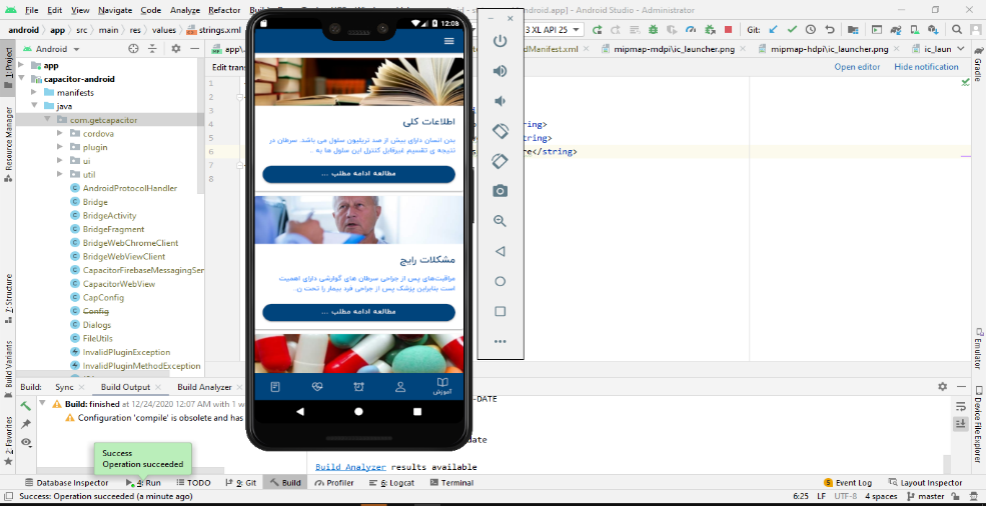
The “Behyar” Android app.

**Figure 2:**
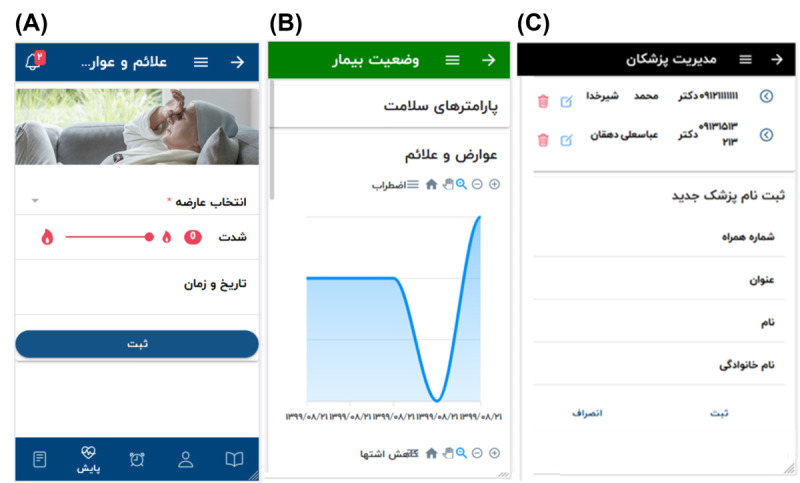
Behyar app. (A) A page in the survivor panel for entering the side effect. The drop-down shown on top is for selecting the side effect. The slider shown with a fire icon is for choosing the intensity of that side effect, and the last input is for selecting the date and time of occurrence. (B) A page in the clinician panel to monitor the side effects of the survivor. The figure shows the intensity of appetite loss on multiple dates. (C) A page in the Admin panel for managing clinicians in the system. The page shows the information of 2 clinicians and a form for adding a new clinician to the system.

The output of the Android-based app was created (Figure 1). The system data items were designed to be flexible so that the specialist could add the required data item to the system if not by default. The client-side app includes 3 panels for survivors, clinicians, and admin, as shown in Figure 2.

### Hardware Design

**Figure 3:**
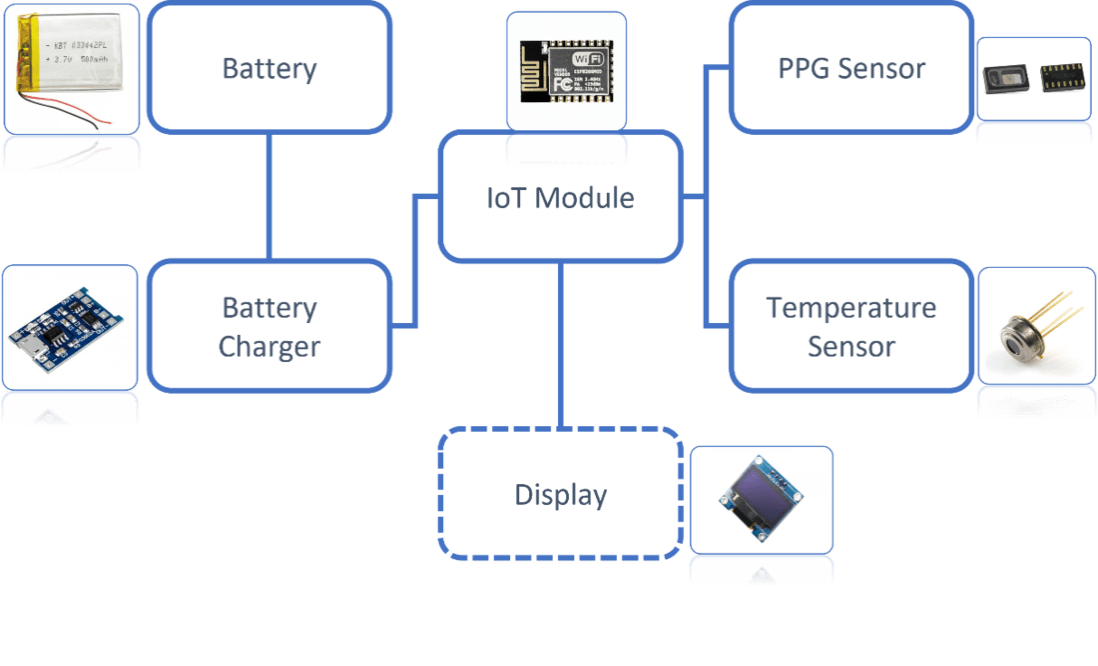
Hardware block diagram of the wristband. PPG: photoplethysmography.

The hardware block diagram of the designed device is shown in Figure 3.

**Figure 4:**
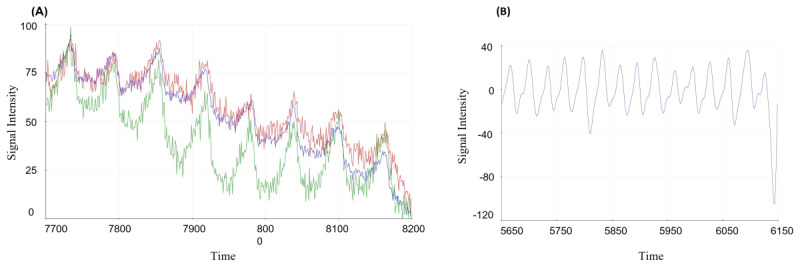
Photoplethysmography (PPG) signal retrieved from the MAX30101 PPG sensor. (A) Raw PPG signal from the wrist. (B) PPG signal after applying a filter.

The MAX30101 PPG signal obtained from the wrist is shown in Figure 4A. The red, blue, and green colors show the PPG signals from RED, IR, and GREED LEDs, respectively. For smoothing the signal obtained from the MAX30101 module, the fast Fourier transform technique was used. The frequencies greater than 4 Hz and less than 0.5 Hz were filtered (Figure 4B).

The prototype of the circuit was developed with modules on the breadboard (Figure S2A in Multimedia Appendix 1), the firmware code was written in the Arduino IDE, and the firmware was uploaded to the Wemos D1 mini development board. The soldering of parts was performed on a printed circuit board (Figure S2B in Multimedia Appendix 1). The assembled hardware is shown in Figure S2C in Multimedia Appendix 1. The hardware’s firmware was developed to have the capability of being upgraded remotely (FOTA: Firmware Over-The-Air).

The enclosure was designed to fit the dimensions of the circuit shown in Figure S3A in Multimedia Appendix 1. After the 3D design of the frame, the 3D prototype of the enclosure was printed. Finally, the wristband was created, as shown in Figure S3B in Multimedia Appendix 1.

The Arduino IDE was used for hardware programming. Altium Designer 2020 (Altium Limited) and SolidWorks 2019 were used for designing the printed circuit board and the frame, respectively.

### System Usability Evaluation

The 4 male experts finally evaluated the current software of the system in terms of usability based on Nielsen’s 10 principles. The experts included cancer surgeons, radiation oncologists, and blood and cancer subspecialists with a mean age of 45 years and an average of 12 years of clinical experience. According to the results presented in Table 1, this system generally gained an average score of 3.8 out of 5 in terms of usability.

**Table 1:** Result of system usability evaluation based on Nielsen’s 10 principles.

Items	Score 1	Score 2	Score 3	Score 4	Score 5	Average score
Visibility of system status (very confusing to very clear)	0	0	1	1	2	4.25
Match between the system and the real world (irrational to very logical)	0	1	2	1	0	3
User control and freedom (this is not possible to this is very convenient)	0	0	0	4	0	4
Consistency and standards (vague to clear)	0	0	1	3	0	3.75
Error prevention (never to always)	0	0	0	4	0	4
Recognition rather than recall (from inappropriate to appropriate)	0	1	0	0	3	4.25
Flexibility and efficiency of use (very inappropriate to very appropriate)	0	1	1	1	1	3.5
Aesthetic and minimalist design (very inappropriate to very appropriate)	0	0	1	1	2	4.25
Recognize, diagnose, and recover from errors (never to always)	0	1	0	2	1	3.75
Help and documentation (inadequate to appropriate)	0	1	1	2	0	3.25

## Discussion

### Principal Findings

In this study, the system for remote monitoring of patients undergoing surgery due to CRC was designed according to the identified priorities. The architecture was considered front end and back end separately for modular designing and creating multiple program versions to run on different platforms [30].

Based on the overall architecture of the system, suitable tools were applied to create the system. ASP.NET Core 3.1 was used because of its open-source, multiplatform capability, and flexibility in development. Security and access levels were defined based on the roles defined in the system and access tokens on the web service.

Client-side software was designed to be hybrid to run on different platforms. PWA and Android software have been used in various fields of health care [31-33]. Secure Sockets Layer protocol was installed on the webserver for communication security. Since the core of the current system’s software is designed as a web service, it is possible to integrate it with other software systems.

Off-the-shelf commercial wristbands with the ability to measure heart rate along with body temperature were not found in the Iranian market at a reasonable price. Thus, the wristband device is designed. The detection of the heartbeat was enabled on the hardware device by PPG technology. PPG technology is a noninvasive technology for measuring various indicators such as heart rate [34] and blood pressure [35]. It is used by a ready-made module (MAX30101) to eliminate noise and reach the signal with higher accuracy.

The findings show that green light has a better PPG signal than red and infrared light due to its greater penetration power in the wrist tissue. This is also mentioned by Fortino and Giampà [35]. The filter (fast Fourier transform algorithm) was used to remove noise, improving signal quality.

It is noteworthy that the price of the sensor selected to measure body temperature following the COVID-19 pandemic and the high demand for this sensor to measure body temperature were about 15 times the price increase, making it difficult to provide.

The use of IoT technologies to design system hardware was considered in this study. The whole system could be considered a Medical Internet of Things system. Medical Internet of Things refers to IoT applications in medicine [36]. The 4 IoT core modules were selected and evaluated for suitability. However, the modules that can communicate via General Packet Radio Services require minimal user intervention (there is no need for a pairing process). Due to the requirements of the electronic components for proper operation, especially in conditions with a weak signal antenna, these modules were not used. The ESP8266MOD module with Wi-Fi capability was selected. This module is suitable for connecting sensors and sending sensor data to the central server [37]. Other modules have also been used in studies. For example, in the study of Onubeze [38], the nRF51822 with the MAX30100 module was used to design a wireless heart rate monitor. IoT-based hardware can measure temperature and the PPG signal. This hardware can also be used to measure blood pressure and blood oxygen saturation [39]. If monitoring physical activity is a priority in other diseases, this feature can be added to the wristband by adding accelerometer and pedometer sensors. An intelligently integrated model of the health care system for cancer care is presented in the Onasanya and Elshakankiri study [40]. This model provides 4 layers of cancer care, hospital, data, and service layer, which are designed hardware that can be used in the cancer care layer.

In this study, the wristband enclosure was designed in SolidWorks software and printed using a 3D printer. Due to the high speed of preparation, 3D printing is recommended as a suitable method for making the prototype.

In this study, web software technologies and mobile apps were used. In similar studies, Mayer et al [41], Cheong et al [42], Keng et al [26], and Miller et al [22] used mobile and web applications were used. In the Maxwell-Smith et al [43] study, there is no reference to the technology applied in the system software.

Concerning the hardware presented in this study, the ability to measure heart rate and body temperature was considered. In the study of Miller et al [22], the health professionals for future apps proposed applying wearable outcome measures for detecting increased heart rate and temperature as the key measures that would be helpful in clinical assessments and remote monitoring of CRC surgery.

In other studies related to the monitoring of patients with cancer, for example, Maxwell-Smith et al [43] and Jonker et al [21] applied a commercially available wearable activity monitor (Fitbit) to monitor physical activity, and in another study by Cheong et al [42], hardware was used to monitor physical activity and heart rate. In Sun et al’s [23] study, commercially available wristband pedometers were used to capture data on daily steps for functional recovery monitoring after major abdominal cancer surgery.

In general, based on the advantages expressed in most studies [21,23,44], novel approaches and technology-based solutions to postoperative assessment based on subjective and objective measures and timely intervention in the surgical oncology setting are beneficial. This could improve long-term outcomes and facilitate providing health services. So the development and evaluation of these systems for various cancer surgeries are recommended.

### Strength and Limitations

The system designed in this research was the first monitoring system designed for CRC survivors in Iran, which could be assumed as the strength of this research.

A major limitation of this study was the initial evaluation of the software’s usability. The evaluation of the proposed system should be performed in multiple aspects with the involvement of more experts and patients. Due to the resource limitations in this study, we decided to limit the study’s scope in the initial usability evaluation. Another limitation is the availability of hardware sensors and modules in the Iranian market, which limits the choice of sensors in the hardware design.

### Conclusions

The results showed that the use of a mobile health app could be used to monitor CRC patients. By including features such as the possibility of changing information items by the expert, the system can be provided with the necessary flexibility in different conditions. Additionally, creating hardware for monitoring vital signs along with system software in terms of creating customization capabilities can help obtain quantitative and qualitative data from patients and survivors to possibly provide better care. From the specialists’ perspective, user interface evaluation of monitoring systems for surgical patients with CRC can achieve an acceptable score. To better understand the usefulness of such systems, in addition to evaluating the user interface, continuous surveys of the system’s effects on indicators such as patients’ quality of life, improving their complications, their nutritional status, and their satisfaction with using the system should be considered.

## Appendix

Multimedia Appendix 1 The system architecture and hardware.

## Abbreviations

CRC: colorectal cancer
IDE: Integrated Development Environment
IoT: Internet of Things
PPG: photoplethysmography
PWA: progressive web application

## Data Availability

The data sets generated and analyzed during this study are available from the corresponding author upon reasonable request.
