# Supplementary material for: Remote Monitoring of Colorectal Cancer Survivors Using a Smartphone App and Internet of Things–Based Device: Development and Usability Study
Source: JMIR Cancer. 2023 Feb 15;9:e42250. doi: 10.2196/42250 (PMC9978953; doi:10.2196/42250)

Multimedia Appendix 1

Figure S1. The general architecture and technologies used in the system development.

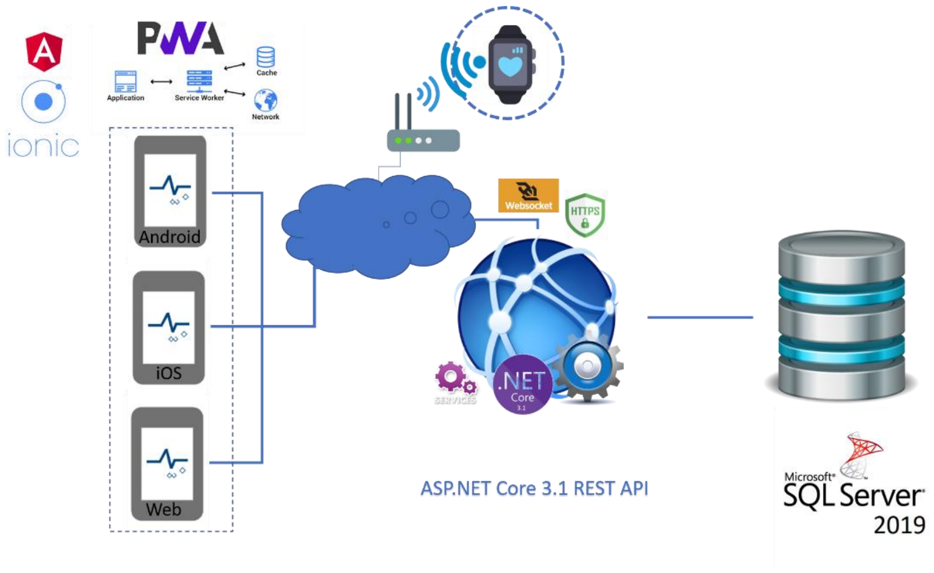

Figure S2. Process of hardware design. (A) Hardware designed on the bread board, (B) soldered parts on the board, and (C) assembled hardware.

(A)

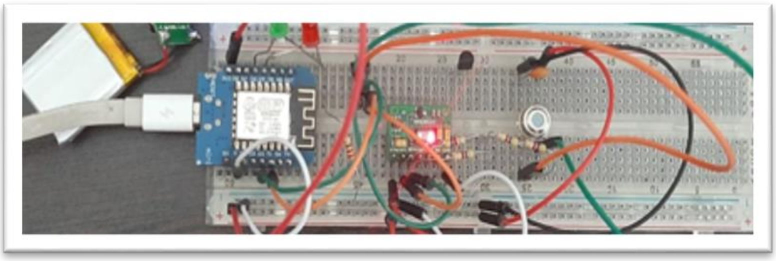

(B)

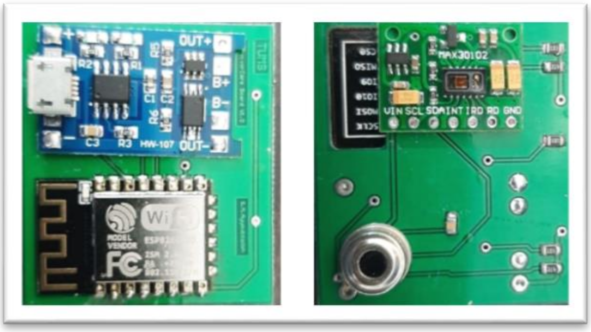

(C)

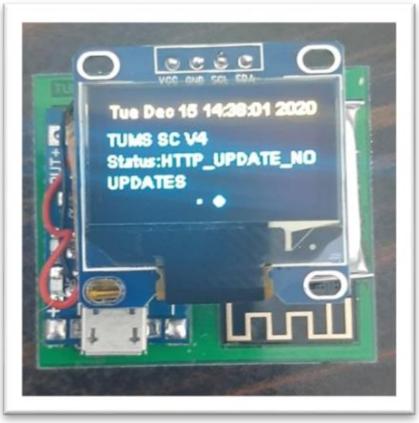

Figure S3. The wristband. (A) Preliminary design of the wristband case and (B) the final wristband.

(A)

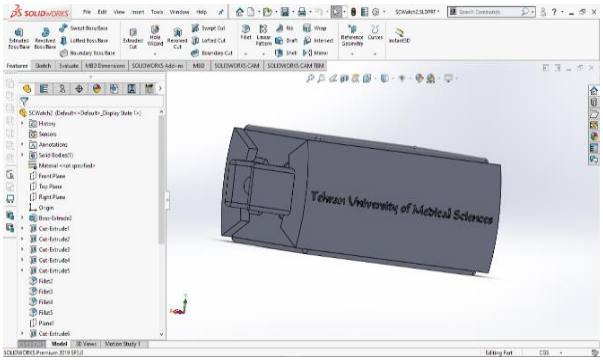

(B)

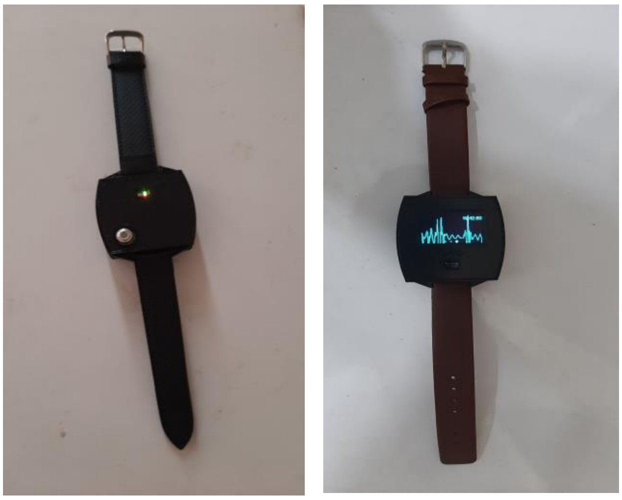

Supplement: Multimedia Appendix 1 [file cancer_v9i1e42250_app1.pdf]
